# The role of macrophages phenotypes in the activation of resolution pathways within human granulosa cells

**DOI:** 10.1186/s12958-022-00983-6

**Published:** 2022-08-10

**Authors:** Thaise S. Martins, Bruno M. Fonseca, Irene Rebelo

**Affiliations:** 1 UCIBIO – Applied Molecular Biosciences Unit, Laboratory of Biochemistry, Department of Biological Sciences, Faculty of Pharmacy, University of Porto, 4050-313 Porto, Portugal; 2 Portugal Associate Laboratory i4HB - Institute for Health and Bioeconomy, Laboratory of Biochemistry, Department of Biological Sciences, Faculty of Pharmacy, University of Porto, 4050-313 Porto, Portugal

**Keywords:** Inflammation, Infertility, Macrophage, Granulosa cells, Cyclooxygenase, Lipoxygenase

## Abstract

**Background:**

Inflammatory state within the ovaries can disrupt normal follicular dynamics, leading to reduced oocyte quality and infertility. How the production of inflammatory mediators generated by macrophages with different gene expression profile (M1 and M2) might activate inflammatory pathways, such as cyclooxygenase-2 (COX-2) and 5-, 12-, and 15-lipoxygenase (LOX), in human granulosa cells (hGCs) remains unclear.

**Methods:**

In this study, we evaluated how M1 and M2 macrophages found in the ovaries affect the functions of hGCs isolated from women undergoing assisted reproductive technology (ART) and human ovarian granulosa COV434 cells. For this purpose, a model of interaction between hGCs and COV434 cells and conditioned media (CMs) obtained from culture of M0, M1 and M2 macrophages was established. We used real-time PCR and western blotting to detect the expression of COX-2 and 5-, 12-, and 15-LOX as biomarkers of oocyte competence.

**Results:**

Our data showed that M2 macrophages with anti-inflammatory characteristics were able to significantly increase the expression of COX-2 in hGCs. We also demonstrated that M1 macrophages with pro-inflammatory characteristics were able to significantly increase the expression of 12-LOX in hGCs. However, there was no observed expression of 5-LOX and no significant alteration in the expression of 15-LOX in hGCs. Regarding COV434 cells, we found that CM from M2 macrophage resulted in an increase in COX-2, 5-LOX and 15-LOX mRNA and protein levels. No expression of 12-LOX by COV434 cells was observed when exposed to CMs from M1 and M2 macrophages.

**Conclusions:**

Our research indicated that the production of pro-resolving mediators by hGCs can, at least in part, reverse the physiological inflammation present in the ovaries.

**Supplementary Information:**

The online version contains supplementary material available at 10.1186/s12958-022-00983-6.

## Introduction

Ovulation displays features that resemble inflammation, which can disrupt the normal follicular dynamics and lead to reduced oocyte quality and infertility [1]. According to the World Health Organization (WHO), infertility is a condition that affects 15% of reproductive-aged couples worldwide, with many requiring the support of assisted reproductive techniques (ART) [2, 3]. Several studies aimed to characterize potential biomarkers of oocyte competence with pregnancy outcomes. Genes expressed by human granulosa cells (hGCs), such as cyclooxygenase-2 (COX-2) and 5-, 12-, and 15-lipoxygenase (LOX), may be useful as effective biomarkers to identify oocytes with high development potential [4, 5].

Ovulation is initiated by the onset of a luteinizing hormone (LH) surge that is accompanied by inflammatory signals, such as inflammatory mediator’s production, blood flow increase, leukocyte infiltration, swelling, and tissue degradation and repair. Pre-ovulatory follicles are comprised of an oocyte surrounded by hGCs, follicular fluid (FF), and a highly vascularized theca cells (TCs) compartment, in which resident leukocytes are found [6].

GCs play a remarkable role in oocyte development, being interconnected with their plasma membrane by gap junctions that sustain them at an arresting stage and transport nutrients. These cells can exhibit distinct phenotypes: mural GCs (MGCs) covering the follicle wall and participating in the physical barrier, estradiol production, and follicle growth; and cumulus cells (CCs) that directly surround the oocyte, providing them with nutrients and protection from harmful signals that can influence their quality [7].

The LH surge activates signalling pathways in GCs of pre-ovulatory follicles associated with inflammation, such as the expression of COX-2 and 5-, 12-, and 15-LOX. These activation leads to the production of inflammatory mediators, such as eicosanoids and specialized pro-resolving mediators (SPMs), which can be found at high levels in human FF impacting on oocyte growth and quality [6–8]. In the ovaries, PGE_2_ contributes to tissue remodelling, follicular wall proteolysis, corpus luteum formation, and regulating vascular changes. All these events are considered as final players of ovulation that coordinate the overall response, which results in the release of the cumulus-enclosed oocyte [6].

In general, inflammation is a defensive response triggered by stimuli that aims to restore homeostasis. The initial response consists of a progression of events from leukocytes recruitment to pro-inflammatory lipids production. A successful acute inflammatory response leads to the elimination of the stimuli and suspension of pro-inflammatory lipids production, initiating the resolution and repair phases with the production of SPMs [9]. The influx of neutrophils is replaced by macrophages that undergo reprogramming from classically activated M1 macrophages to alternatively activated M2 macrophages [10].

Macrophages are among the predominant immune cells in the acute inflammatory response and ovulation, being found at high levels in FF [11]. The molecular pathways in which these cells are involved in regulation ovulation are not yet fully elucidated. However, evidence suggests that macrophages are implicated in leukocyte recruitment, secretion of immune mediators, breakdown of the extracellular matrix component, and regulation of tissue remodelling after ovulation [12]. Since macrophages act as drivers and regulators of diseases, therapeutic strategies that either decrease the amount and function of M1 or increase M2 activity are undergoing extensive pre-clinical and clinical research [13].

However, uncontrolled or unsuccessful immune responses can lead to continued recruitment of inflammatory cells. The persistence of tissue damage results in organ dysfunction and, consequently, the establishment of chronic inflammatory diseases [14]. An enhanced understanding of the role of inflammation in ovulation and the control of this process may lead to exploiting this knowledge to treat anovulatory infertility.

Since the crosstalk between hGCs and macrophages shows issues relevant to the production of key factors during oocyte development, this relationship requires further investigation. Therefore, the present study aimed to simulate the ovarian microenvironment and investigate how the production of pro- and anti-inflammatory mediators generated by M1 and M2 macrophages, respectively, might activate inflammatory pathways in GCs isolated from women undergoing in vitro fertilization (IVF)/intracytoplasmic sperm injection (ICSI) techniques.

## Materials and methods

### Materials

All reagents and solvents were used of analytical grade. Dulbecco’s modified eagle medium F-12 (DMEM/F-12), Roswell park memorial institute 1640 (RPMI-1640), methylthiazolyldiphenyl-tetrazolium bromide (MTT), and dimethyl sulfoxide (DMSO), lipopolysaccharide (LPS), were purchased from Sigma–Aldrich Co. (St. Louis, MO, USA). Phorbol 12-myristate 13-acetate (PMA) was obtained from Santa Cruz Biotechnology. Penicilin and streptomycin (AB-AM) were bought from Grisp (Porto, Portugal). Trypsin and ethylenediaminetetraacetic acid (EDTA) were obtained from Gibco/Invitrogen Co. (Carlsbad, CA, USA). Fetal bovine serum (FBS) came from Biochrom and percoll from GE Healthcare (Buckinghamshire, UK). Recombinant human interferon-γ (IFN-γ) was purchased from R&D Systems and recombinant human interleukin-13 (IL-13) from Biotechne. Dibutylphthalate polystyrene xylene (DPX) was bought from VWR-Prolabo (Radnor, PA, USA). THP-1 cells (#88,081,201) and COV434 cells (#07,071,909) were purchased from the European Collection of Authenticated Cell Cultures (ECACC). Cell culture flasks were obtained from Sarstedt (Nümbrecht, Germany) and all other plastic materials used in cell culture were from Falcon (Tewksbury, MA, USA) and Nerbe plus (Winsen, Germany).

### Methods

#### Study design

FF samples containing hGCs retrieved from women undergoing ovarian stimulation for ART were harvested with their agreement at Unidade de Medicina da Reprodução Dra. Ingeborg Chaves-Centro Hospitalar de Vila Nova de Gaia/Espinho, from January 2021 to April 2022. A group of 32 patients was enrolled in this study, with a mean of 32 years of age and 21.5 kg/m^2^ of body mass index (BMI). Inclusion criteria were women presenting male factor associated to infertility. Women with endometriosis, polycystic ovary syndrome (PCOS), tubal factor, and idiopathic factor, as well as women with infertility associated to hormonal factors, were excluded. Data regarding the oocyte number, maturity, fertilization rate, and clinical pregnancy were collected. All procedures were conducted in accordance with the Declaration of Helsinki, endorsed by the local ethics committee and approved by Comissão de Proteção de Dados (Proc. no. 764/2017).

#### Collection and isolation of primary human granulosa cells

Ovarian stimulation was triggered with different dosages of gonadotropins, follicle stimulating hormone (FSH) and/or LH, for multifollicular development. Then, the final stimulus was performed by a single administration of human chorionic gonadotropin (hCG) or gonadotropin-releasing hormone (GnRH) agonist 36 h before the oocyte retrieval. For ART performance, the FF consisting of oocyte and hGCs was aspirated and the oocyte was isolated for further fertilization. The remaining FF was transferred to 100 mL polypropylene tubes and stored at 37ºC for a maximum of 2 h until sample processing. hGCs were isolated and cultured independently according to the protocol previously published by Moreira-Pinto et al. [15], with some modifications. Briefly, FF samples were centrifuged at 350 g at 10 °C for 10 min. The cell pellet was collected and slowly added to a Percoll:PBS (1:1) density gradient. The samples were then centrifuged at 900 g at 10 °C for 20 min. hGCs present at the interface of Percoll:PBS and FF gradient were collected into a new tube, in which DMEM/F12 10% FBS was added and centrifuged at 350 g at 10 °C for 5 min to wash cells. The supernatant was discarded and the hGCs were resuspended in DMEM/F12 10% FBS.

#### Human granulosa cell line COV434 culture conditions

Human ovarian granulosa COV434 cells originally derived from a solid primary tumor were used as model of GCs and cultured according to specific indications from ECACC [16]. COV434 cells were cultured in DMEM/F12 10% FBS medium supplemented with Ɩ-glutamine (2 mM), 1% AB-AM, and 10% FBS at 37 °C with 5% CO_2_ atmosphere. COV434 cells were maintained at a minimum density of 2.0 × 10^4^ cells/mL and were trypsinized using 0.25% trypsin/EDTA (1 mM) upon reaching 4.0 × 10^4^ cells/mL.

#### Human monocyte cell line THP-1 culture conditions

Human acute monocytic leukemia THP-1 cells derived from the peripheral blood were used as model of human monocytic cells and cultured according specific indications from ECACC [17]. THP-1 cells were cultured in RPMI 1640 medium supplemented with Ɩ-glutamine (2 mM), 1% AB-AM, and 10% FBS at 37 °C with 5% CO_2_ atmosphere. THP-1 cells were maintained at a minimum density of 3.0 × 10^5^ cells/mL and were passaged upon reaching 8.0 × 10^5^ cells/mL.

#### THP-1 derived macrophages culture conditions

THP-1-derived macrophages were obtained following a previous procedure described by Fonseca et al. [18], with some modifications. Firstly, THP-1 cells were seeded at a density of 5.0 × 10^5^ cells/well in a 24-well plate and stimulated with PMA (25 ng/mL) for 48 h. After PMA exposure, the medium was discarded and the cells were washed with PBS, followed by a further incubation in fresh medium for 24 h as a resting period. Then, macrophages were differentiated into M1 and M2 phenotypes by incubation with IFN-γ (20 ng/mL) + LPS (10 pg/mL) and IL-13 (20 ng/mL), respectively, for 24 h. After macrophages polarization, the differentiation factors were removed and the cells were washed with PBS, followed by a new incubation in fresh medium for 72 h, initiating cytokines production. After 72 h, macrophage supernatants were collected, centrifuged at 300 g at 10ºC for 5 min to remove cell debris and then stored at -80ºC until use.

#### Interaction model between cell cultures

To evaluate how macrophage phenotypes found in the ovaries affect the GCs functions, an interaction model was established between hGCs and COV434 cells and conditioned media (CMs) obtained from culture of M0, M1 and M2 macrophages [18]. hGCs were seeded in a 6-well plate (1.5 × 10^6^ cells/well) and 24-well plate (7.0 × 10^5^ cells/well) and COV434 cells were also seeded in a 6-well plate (8.0 × 10^5^ cells/well) and 24-well plate (3.5 × 10^5^ cells/well) in DMEM/F12 10% FBS, and incubated for 24 h for cell adhesion. After incubation, the medium was removed, the CMs obtained from culture of M0, M1 and M2 macrophages were added in a 6-well plate (2000 μL) and 24-well plate (500 μL), and incubated for 72 h at 37 °C with 5% CO_2_ atmosphere.

#### Cell viability

hGCs and COV434 cells were seeded at densities of 7.5 × 10^4^ and 5.0 × 10^4^ cells/well, respectively, in a transparent 96-well plate. After 24 h, the medium was removed, CMs obtained from culture of M0, M1 and M2 macrophages were added to a final volume of 200 μL, and incubated for 72 h at 37 °C with 5% CO_2_ atmosphere. MTT assay conditions were adapted from the work by Castelôa et al. [19]. Briefly, at the end of incubation time, an MTT solution was added (0.5 mg/mL) and the plate was incubated for another 3 h at 37ºC with 5% CO_2_ atmosphere. After incubation, the medium was removed and a solution of DMSO/isopropanol (3:1) was added to dissolve the purple MTT formazan crystals. The plate was gently shaken at 70 rpm for 15 min to dissolve the crystals, which were quantified spectrophotometrically at 540 nm using a microplate reader (BioTek Synergy HTX Multi-Mode).

#### Cell morphology analysis

After 72 h of interaction between CMs obtained from culture of M0, M1 and M2 macrophages, morphological abnormalities in hGCs and COV434 cells were evaluated by Giemsa stain [19]. hGCs and COV434 cells were seeded in a 24-well plate with round coverslips at densities of 7.0 × 10^5^ and 3.5 × 10^5^ cells/well, respectively. After adherence, 500 μL of CMs of M0, M1 and M2 were added to the cells and incubated for 72 h at 37 °C with 5% CO_2_ atmosphere. After incubation, the CMs were removed, cells were washed twice with PBS, and fixed with cool methanol for 10 min at room temperature (RT). Then, the methanol was removed, cells were washed again twice with PBS, Giemsa solution in H_2_0 (1:10) was added, and the cells were incubated for a further 30 min. After incubation, the cells were washed several times with tap water and the coverslips were removed, dried, and mounted with DPX medium. Cells were observed under the Eclipse CI microscope (Nikon, Japan).

#### RNA extraction, cDNA synthesis and Real-Time Polymerase Chain Reaction (RT-PCR)

For macrophages derived from THP-1 differentiation (*n* = 3, in triplicate) and hGCs and COV434 cells experiments (*n* = 3, in duplicate), cells were lysed with TripleXtractor reagent (#GB23.0050; GRiSP Research Solution, Porto, Portugal) to isolate total RNA according to the manufacturer’s protocol. The concentration and purity of the isolated RNA were measured using a NanoDrop ND-1000 Spectrophotometer (NanoDrop Technologies, Inc., Wilmington, DE, USA) and samples were stored at -20ºC prior to cDNA synthesis. The isolated RNA was converted to cDNA using the Xpert cDNA Synthesis Mastermix (#GK81.0100; GRiSP Research Solution, Porto, Portugal) according to the manufacturer's protocol. Samples were diluted in H_2_O treated with diethyl pyrocarbonate to a final volume of 40 μL and then stored at -20ºC or immediately used as a template in RT-PCR. The cDNA was amplified with specific primers (Table 1), using Xpert Fast SYBR Mastermix (#GE20.2501; GRiSP Research Solution, Porto, Portugal) in Bio-Rad Real-Time PCR Detection System (Bio-Rad Laboratories, USA), according to the manufacturer’s protocol. RT-PCR conditions were, in all cases, initiated with a denaturation step at 95 °C for 3 min, followed by up to 40 cycles of denaturation, annealing, and primer extension. The fold change in gene expression was calculated using the 2^−ΔΔCt^ method and normalized with the housekeeping gene glyceraldehydes 3-phosphate dehydrogenase (GAPDH).

**Table 1:** Sequences of transcript primers and annealing temperatures for RT-PCR

mRNA Target	Primer Sequence: 5’-3’	Annealing temperature	Acession Number^a^	Ref
GADPH	Forward-GGATGATGTTCTGGAAGAGCC	55ºC	-	[15]
	Reverse-AACAGCCTCAAGATCATCAGC			
IL-1β	Forward-ATGATGGCTTATTACAGTGGCAA	60ºC	-	[20]
	Reverse-GTCGGAGATTCGTAGCTGGA			
TNF-α	Forward-GCTGCACTTTGGAGTGATCG	60ºC		
	Reverse-GTGTGCCAGACACCCTATCT			
CCL18	Forward-TGCATTGCAGCGTCATCTTG	57ºC	NM_002988.4	-
	Reverse-GAGTCCCATCTGCTATGCCC			
CCL22	Forward-AGGGCCAGGGGACATCTAAT	57ºC	NM_002990.5	-
	Reverse-GAGATCTGTGCCGATCCCAG			
COX-2	Forward-GTTCCACCCGCAGTACAGAA	58ºC	NM_000963.4	-
	Reverse-AGGGCTTCAGCATAAAGCGT			
5-LOX	Forward-CATGCCCAGGAACAGCTCGTT	57ºC	-	[21]
	Reverse-AGTCCTCAGGCTTCCCCAAGT			
12-LOX	Forward-GCCCAAAGCTGTGCTAAACC	58ºC	NM_000697.3	-
	Reverse-TGGGGGAGGAAATAGAGCCT			
15-LOX	Forward-CCTTCGTCCTCCAAACCTGT	57ºC	NC_000017.11	-
	Reverse-GCTCGGATGTGGGTAGTGAC			

^a^Primers have been designed in-house by using Primer Blast from NIH

#### Western blotting

For hGCs and COV434 cells experiments (*n* = 3 for each target), CMs obtained from culture of M0, M1 and M2 macrophages were removed, cells were washed twice with PBS, lysed with lysis buffer (Tris–HCl 20 mM, NaCl 100 mM, EDTA 1 mM, and Triton X-100 1%) containing a protease inhibitor cocktail (#P8340; Sigma–Aldrich Co., St. Louis, MO, USA), and incubated on ice for 30 min. After incubation, cell extracts were harvested, frozen/thawed three times and centrifuged at 14000 g at 4ºC for 10 min. The supernatant was collected and the protein concentration was quantified by Bradford assay. Samples (5–40 μg) were subjected to 10% SDS–PAGE and proteins were transferred to nitrocellulose membranes in a turbo transfer system (Trans-Blot®, Bio-Rad). After blocking in 5% non-fat milk for 1 h at RT, the membranes were incubated with the primary antibodies, COX-2 (#sc-23984, Santa Cruz Biotechnology), ALOX5 (#MA5-26,829, ThermoFisher Scientific), and ALOX12 (#MA5-26,911, ThermoFisher Scientific) diluted at 1:500, and 15-LOX (#sc-133085, Santa Cruz Biotechnology) diluted at 1:250. After incubation, primary antibodies were removed, membranes washed with PBS-Triton, and incubated with the secondary antibodies, peroxidise-linked anti-goat (#sc-2354, Santa Cruz Biotechnology) and anti-mouse (#sc-516102, Santa Cruz Biotechnology) both diluted at 1:2500 for 1 h at RT. Then, secondary antibodies were removed, membranes were washed again with PBS and PBS-Triton, and exposed to the chemiluminescent substrate. Immunoreactive bands were visualized by the ChemiDocTM Touch Imaging System (BioRad, Laboratories Melville, NY, USA). Membranes were then stripped and incubated with β-Actin (sc-47778, Santa Cruz Biotechnology) diluted at 1:500 to control loading variation.

#### Statistical analysis

Statistical analysis was performed employing One-Way ANOVA, followed by post hoc Bonferroni’s test to compare the individual means. Results are displayed graphically as means ± SEM (standard error mean). A *p* value < 0.05 was considered statistically significant. Statistical analysis was performed using GraphPad PRISM (version 8.0; GraphPad Software, Inc., San Diego, CA).

## Results

### M0, M1 and M2 macrophages derived from THP-1 differentiation

Following THP-1 monocytes stimulation with PMA (25 ng/mL) for 48 h, it was possible to confirm the differentiation of monocytes into resting macrophages (M0) by checking cell morphology under phase contrast microscopy.

**Fig. 1:**
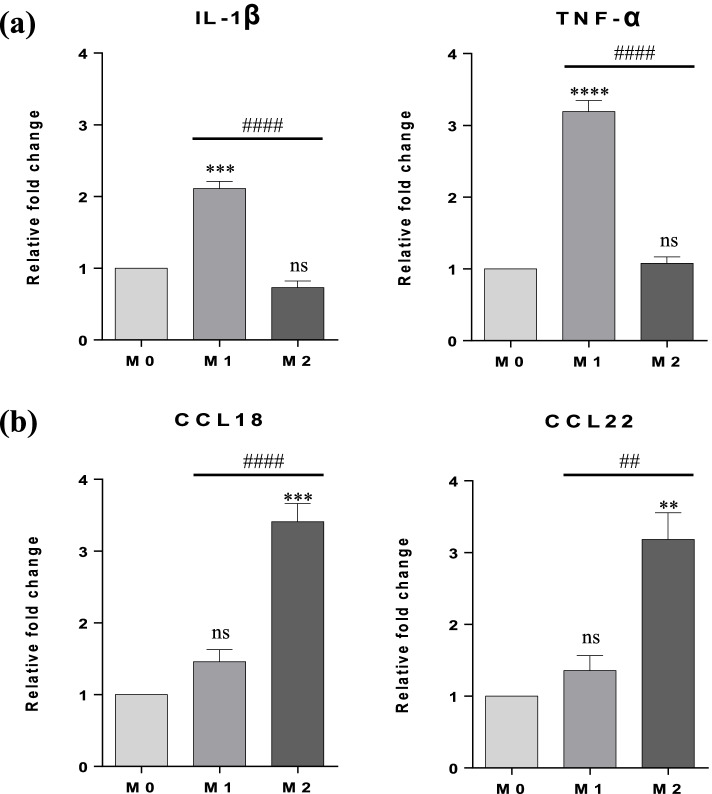
Expression of M1 and M2 markers after 24 h of polarization with differentiation factors. (**a**) mRNA expression levels of M1 markers (IL-1β and TNF-α). (**b**) mRNA expression levels of M2 markers (CCL18 and CCL22). The relative fold change in gene expression was calculated using 2^−ΔΔ^^Ct^ method and normalized with the housekeeping gene GAPDH. Results are expressed as means ± SEM (*n* = 3, in triplicate). Statistical analysis was based on one-way ANOVA, followed by a post hoc Bonferroni’s multiple comparisons test. ns: not significant. ** *p* < 0.01, *** *p* < 0.001 or *p* < **** 0.0001: significantly different from the control (M0). ## *p* < 0.01, ### *p* < 0.001 or *p* < #### 0.0001: significantly different between M1 versus M2

After obtaining resting macrophages (M0), the polarization of M1 with IFN-γ (20 ng/mL) + LPS (10 pg/mL) and M2 with IL-13 (20 ng/mL) for 24 h, was confirmed in mRNA levels by RT-PCR measuring the expression of specific marker genes for M1 (IL-1β and TNF-α) and M2 (CCL18 and CCL22). No expression of any M2 marker was highlighted in M1 and vice versa (Fig. 1).

### Effects of conditioned media obtained from culture of M1 and M2 macrophages in hGCs and COV434 cells viability

**Fig. 2:**
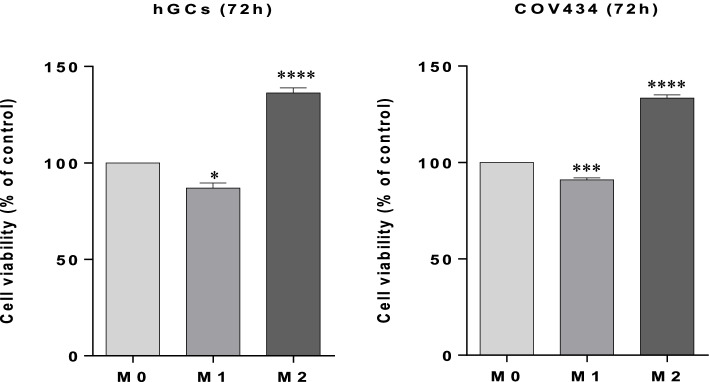
Effects of conditioned media obtained from culture of M1 and M2 macrophages in hGCs and COV434 cells viability. MTT assay after interaction between CMs obtained from culture of M1 and M2 macrophages and hGCs and COV434 cells at 72 h. Results are compared to the control (M0) and expressed as means ± SEM (at least *n* = 3, in triplicate). Statistical analysis was based on one-way ANOVA, followed by a post hoc Bonferroni’s multiple comparisons test. ns: not significant. * *p* < 0.05, *** *p* < 0.001 or *p* < **** 0.0001: significantly different from the control (M0)

After 72 h of interaction, it was possible to observe that in both hGCs and COV434 cells, the CMs obtained from culture of M1 and M2 macrophages affected significantly the cell viability when compared to the resting macrophages (M0) considered as control (Fig. 2).

### Effects of conditioned media obtained from culture of M1 and M2 macrophages in hGCs and COV434 cells morphology

**Fig. 3:**
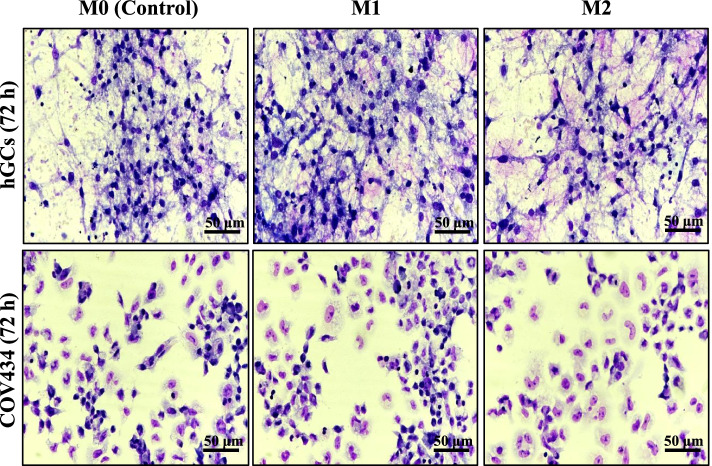
Effects of conditioned media obtained from culture of M1 and M2 macrophages in hGCs and COV434 cells morphology. Cell morphology was analyzed after 72 h using Giemsa stain. Untreated cells containing only the media (RPMI 1640 10% FBS) were used as control (M0). Results are shown from single representative of three independent experiments

No morphological changes could be noted in hGCs and COV434 cells after 72 h exposed to CMs obtained from culture of M1 and M2 macrophages (Fig. 3).

### Effects of conditioned media obtained from culture of M1 and M2 macrophages on COX-2 and 5-, 12-, and 15-LOX expression in hGCs and COV434 cells

**Fig. 4:**
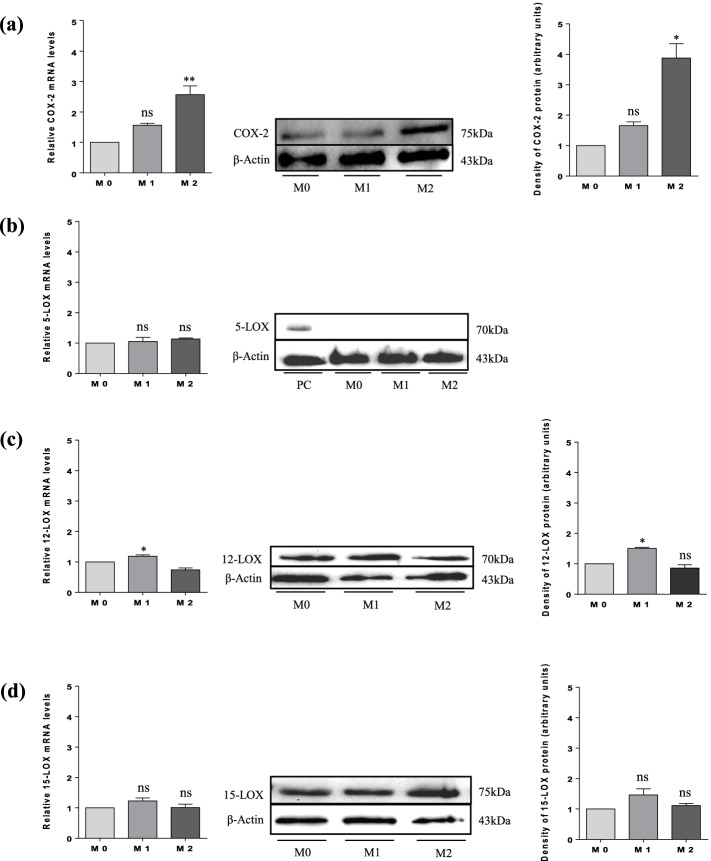
Expression of COX-2 and 5-, 12-, and 15-LOX in hGCs by mRNA and protein levels. (**a**) COX-2 expression; (**b**) 5-LOX expression; (**c**) 12-LOX expression; and (**d**) 15-LOX expression. Results are compared to the control (M0) and expressed as means ± SEM. For PCR results (*n* = 3, in duplicate) and for western blotting (a single representative of three independent experiments). Statistical analysis was based on one-way ANOVA, followed by a post hoc Bonferroni’s multiple comparisons test. ns: not significant. * *p* < 0.05, ** *p* < 0.01

**Fig. 5:**
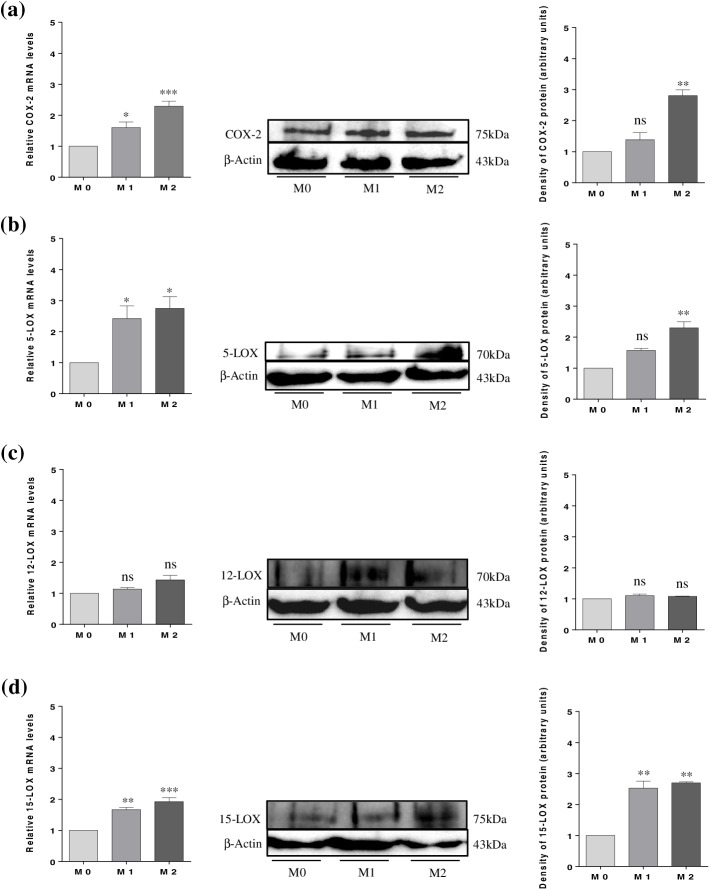
Expression of COX-2 and 5-, 12-, and 15-LOX in COV434 by mRNA and protein levels. (**a**) COX-2 expression; (**b**) 5-LOX expression; (**c**) 12-LOX expression; and (**d**) 15-LOX expression. Results are compared to the control (M0) and expressed as means ± SEM. For PCR results (*n* = 3, in duplicate) and for western blotting (a single representative of three independent experiments). Statistical analysis was based on one-way ANOVA, followed by a post hoc Bonferroni’s multiple comparisons test. ns: not significant. * *p* < 0.05, ** *p* < 0.01, *** *p* < 0.001

After confirmation of THP-1 differentiation, an interaction model between CMs obtained from culture of M1 and M2 macrophages and hGCs and COV434 cells was established. After 72 h of incubation, cells were lysed for mRNA and protein extraction and checking the COX-2 and 5-, 12-, and 15-LOX expression in hGCs (Fig. 4) and COV434 cells (Fig. 5), by mRNA levels using RT-PCR and protein levels using western blotting.

## Discussion

Macrophages are the most dominant ovarian leukocytes that can be found in the theca layers of growing follicles and in human FF [22]. Over the last few decades of investigation, it is becoming increasingly evident that macrophages are phenotypically and functionally heterogeneous. In fact, macrophages display high degrees of plasticity in their roles when exposed to various environments. M1 macrophages are differentiated by Th1 cytokines, such as IFN-γ, or by recognition of LPS, producing high levels of pro-inflammatory cytokines (TNF-α and IL-1β). Functionally, the pro-inflammatory M1 macrophages are implicated in the clearance of pathogens in the course of infection by generating reactive oxygen/nitrogen species (ROS/RNS), which can induce tissue damage. In contrast, M2 macrophages are differentiated by Th2 cytokines, such as IL-4 and/or IL-13, secreting pro-resolving mediators (CCL18, CCL22, and SPMs). Functionally, pro-resolving M2 macrophages show the ability to phagocytose and promote tissue remodelling and wound healing. The heterogeneous and dynamic nature of macrophages in the ovaries implies their proactive involvement in ovarian homeostasis and hormonal control [23].

Considering that M1 and M2 macrophages are associated with inflammatory processes, macrophages polarization may be an important aspect of their interaction with hGCs. Thus, the first aim of this study was to obtain M1 and M2 macrophages phenotypes from the THP-1 human monocyte cell line. The THP-1 is an immortalized cell line isolated from the peripheral blood of a 1-year-old male patient with monocytic leukemia. Resting THP-1 cells retain most of the inflammatory monocytes signalling pathways and exhibit the ability to differentiate into macrophages when stimulated with PMA. For these reasons, this cell line was chosen as an appropriate cell model for obtaining macrophages derived from human monocyte [24].

However, although the THP-1 cell line is widely recognized as a suitable model for assessing macrophage functions and responses to foreign stimuli in vitro, there is currently no standardized protocol for the differentiation of THP-1 monocyte into macrophages using PMA. The lack of a standard protocol has a significant impact on the interpretation of results and comparison of studies. The differentiation protocol used in this study was based on the one previously published by our research group. We initiated THP-1 differentiation by exposing the cells to PMA (25 ng/mL) for 48 h, followed by a 24 h resting period [18]. Differentiation of monocytes into resting M0 was confirmed by observation that macrophage-like cells adhered to tissue culture flask, adopting a stellate morphology. After that, resting M0 were primed for 24 h with fresh medium supplemented with IFN-γ (20 ng/mL) + LPS (10 pg/mL) to differentiate into M1 and by IL-13 (20 ng/mL) for M2. Transcriptional markers previously used to characterize distinct subsets of macrophages were identified in the literature and used in the present study [24, 25]. As stated earlier, a distinctive hallmark of M1 polarization is the high production of pro-inflammatory cytokines, while M2 polarization regulates the production of pro-resolving mediators. Thus, M1 and M2 macrophages have been shown to be distinguished by distinct chemokine arrays. Studies conducted in ex vivo human systems reported that IL-1β and TNF-α genes were expressed in the M1 macrophages [26]. Our results showed a very similar array of up-regulated genes in M1 macrophages, suggesting a successful differentiation. In turn, the chemokines CCL18 and CCL22 are categorized according to the M2 state [25, 26]. Our findings show that M2 macrophages expressed all the chemokines mentioned above (Fig. 1).

Inflammatory conditions can severely disrupt normal ovarian function and oocyte quality. In vitro models that adequately simulate the ovarian microenvironment may provide a helpful tool to study the mechanisms by which pro-inflammatory M1 and pro-resolving M2 macrophages may affect the hGCs functions. At this point, we established an interaction model between CMs obtained from culture of M1 or M2 macrophages and GCs to mimic the ovary in inflamed states. This model of interaction has long been widely used to study fundamental cellular interactions of any kind. These systems are highly relevant for drug discovery as they provide a more representative in vivo model of human tissue than animal models [27]. Culture models using CMs obtained from another cell type incorporate a physical barrier between cell types, allowing only signalling through the cell secretome (CS). The CM is commonly employed, where the medium is used first to culture one cell type and then transferred to the second cell type. The CM is constituted by the CS of the first cell type that contains soluble factors and can affect the behaviour of the second cell type in a positive and/or negative way [28].

In the culture model adopted in this study, we decided to use only primary human GCs isolated from women who had male factor as a cause of infertility. Exclusion criteria for women with endometriosis or PCOS, for example, were based on eliminating cells obtained from inflammatory-associated pathologies and, therefore, investigating only the contribution of macrophages to physiological ovarian inflammation. An immortalized hGCs line could be helpful in studying many of the processes that are implicated in human follicle development [29]. We chose to employ the COV434 cell line due to its ability to synthesize estradiol from FSH stimulation, presence of specific markers of apoptosis enabling the induction of follicular atresia, and capacity to form intercellular connections with cells surrounding an oocyte [29, 30].

Regarding cell viability, we observed that CMs obtained from culture of M1 and M2 macrophages affected the viability of both hGCs and COV434 cells, when compared to the resting macrophage (M0), which was used as a control. However, no morphological changes were observed in both cells. As seen in Fig. 2, when hGCs and COV434 cells are exposed to CM of M1 macrophages, a significant decrease in cell viability occurs, suggesting that the pro-inflammatory environment may be influencing cells’ viability. However, it is possible to notice a greater significant difference in the decrease of hGCs viability (*p* < 0.05) in comparison to COV434 (*p* < 0.001), which in part can be explained by the fact that hGCs are originated from a previous inflammatory environment, such as ovaries. Furthermore, it is known that pro-inflammatory M1 macrophages can produce ROS/RNS, which can induce apoptosis in hGCs [31]. However, this observation requires further investigation. Thus, the pro-inflammatory environment can affect the ovarian oxidative balance. Regarding cell viability when hGCs and COV434 cells are exposed to the CM of M2 macrophages, it can be observed that it is affected in a similar statistical proportion for both cells (*p* < 0.0001) when compared to the M0 control, suggesting that the anti-inflammatory environment provided by M2 macrophages can, to some extent, contribute to cell proliferation.

In the present study, the interaction between CM obtained from culture of M2 macrophages and hGCs and COV434 cells for 72 h induced a significant increase in mRNA and protein levels of COX-2. However, no significant increase was observed when cells were exposed to CM obtained from culture of M1 macrophages (Figs. 4a and 5a). Narki et al. [32] previously reported that the expression of COX-2 in GCs isolated from women undergoing ART was induced by the pro-inflammatory cytokine IL-1β, contrasting our results. From these findings, it is possible to suggest that the induction of COX-2 expression by the CM of M2 macrophages may increase the production of pro-resolving mediators by hGCs and COV434 cells, leading to the resolution of inflammation.

Feldam et al. [33] demonstrated for the first time the expression of 5-, 12-, and 15-LOX in GCs isolated from women undergoing ART through the characterization of specific products derived from the metabolism of arachidonic acid (AA), such as 5-, 12-, and 15-hydroxyeicosatetraenoic acids (HETEs). In a recent study, Zhang et al. [8] reported that the pro-resolving mediator resolvin E1 (RvE1) improved oocyte quality by decreasing apoptosis rate of CCs and increasing cell viability and proliferation. Considering that COX-2 and LOXs, both involved in RvE1 production, when stimulated can produce pro-inflammatory and pro-resolving mediators, we also investigated their expression in hGCs and COV434 cells by CMs of M1 and M2 macrophages. From our data, it is possible to observe that neither CM of M1 nor that of M2 macrophages were effective in inducing 5-LOX expression in hGCs, both in terms of RNA and protein levels, when compared to the positive control (PC, placenta homogenate) (Fig. 4b). Regarding COV434 cells, a significant increase in 5-LOX mRNA levels is observed when exposed to CMs of M1 and M2 macrophages. However, the same was not observed according to the results of western blotting, where a significant increase in 5-LOX protein expression was observed only when the cells were exposed to the CM of M2 macrophages (Fig. 5b). Concerning 12-LOX expression, a significant increase in its expression is only observed when hGCs are exposed to the CM of M1 macrophages (Fig. 4c). However, 12-LOX expression is not observed when COV434 cells are exposed to CMs of M1 and M2 macrophages (Fig. 5c). These results can be explained, at least to some extent, by the fact that the hGCs are originated from an inflammatory environment per se. Thus, increased expression of 12-LOX may be a compensatory way in which cells produce pro-resolving mediators to overcome inflammation. Finally, CMs of M1 and M2 macrophages, despite 72 h of interaction with hGCs, were not able to significantly affect the 15-LOX expression when compared to control (M0) (Fig. 4d). This result contrasts with the one published by Liao et al. [34], in which the authors demonstrated that 15-LOX expression was up-regulated in GCs isolated from women with PCOS. In regard to COV434 cells, a significant increase in 15-LOX expression is observed when these cells are also exposed to CMs of M1 and M2 macrophages (Fig. 5d).

## Conclusions

In conclusion, it can be inferred that M2 macrophages with anti-inflammatory characteristics were able to significantly influence the expression of COX-2 and 5-, 12-, and 15-LOX in hGCs. Therefore, it is possible to suggest that the production of pro-resolving mediators by hGCs can, at least in part, reverse the physiological inflammation present in the ovaries. As far as we know, this model of interaction between CMs obtained from culture of M0, M1 and M2 macrophages and hGCs and COV434 cells that aims to investigate the expression of COX-2 and 5-, 12-, and 15-LOX had not been investigated earlier, which enabled us to contribute with new findings related to the role of differentiated macrophages found in the ovaries. However, the extent to which pro-resolving concentrations affect oocyte quality, maturation, fertilization potential, and embryonic development is unclear and requires further investigation.

## Supplementary Information

**Additional file 1:** **Supplementary figures4 and 5.** Uncropped gel from western blots shown in themain figures of the manuscript. Dotted line represents indirect co-culture ofconditioned media of M0, M1 and M2 with hGCs (figure 4) and COV434 (figure5).

## Data Availability

All data generated through this study are included in this article.

## Abbreviations

AA: Arachidonic Acid
ART: Assisted Reproductive Technology
BMI: Body Mass Index
CC: Cumulus Cell
CM: Conditioned Media
COX: Cyclooxygenase
CS: Cell Secretome
DMEM: Dulbecco’s Modified Eagle Medium
DMSO: Dimethyl Sulfoxide
DPX: Dibutylphthalate Polystyrene Xylene
ECACC: European Collection of Authenticated Cell Cultures
EDTA: Ethylenediaminetetraacetic Acid
FBS: Fetal Bovine Serum
FF: Follicular Fluid
FSH: Follicle Stimulating Hormone
GAPDH: Glyceraldehydes 3-Phosphate Dehydrogenase
GnRH: Gonadotropin-Releasing Hormone
hCG: Human Chorionic Gonadotropin
HETE: Hydroxyeicosatetraenoic Acid
hGC: Human Granulosa Cell
ICSI: Intracytoplasmic Sperm Injection
IFN-γ: Interferon-γ
IL: Interleukin
IVF: In Vitro Fertilization
LH: Luteinizing Hormone
LOX: Lipoxygenase
LPS: Lipopolysaccharide
MGC: Mural Granulosa Cell
MTT: Methylthiazolyldiphenyl-tetrazolium bromide
PCOS: Polycystic Ovary Syndrome
PGE_2_: Prostaglandin E_2_
PMA: Phorbol 12-Myristate 13-Acetate
ROS/RNS: Reactive Oxygen/Nitrogen Species
RPMI: Roswell Park Memorial Institute
RT: Room Temperature
RT-PCR: Real-Time Polymerase Chain Reaction
RvE1: Resolvin E1
SPM: Specialized Pro-resolving Mediator
TC: Theca Cell
TNF-α: Tumor Necrosis Factor-alpha
WHO: World Health Organization
