# Supplementary material for: The role of macrophages phenotypes in the activation of resolution pathways within human granulosa cells
Source: Reprod Biol Endocrinol. 2022 Aug 10;20:116. doi: 10.1186/s12958-022-00983-6 (PMC9364504; doi:10.1186/s12958-022-00983-6)
Supplement: Supplementary file 1 — Additional file 1: Supplementary figures4 and 5. Uncropped gel from western blots shown in themain figures of the manuscript. Dotted line represents indirect co-culture ofconditioned media of M0, M1 and M2 with hGCs (figure 4) and COV434 (figure5). [file 12958_2022_983_MOESM1_ESM.docx]

**Supplementary information file**

**The role of macrophages phenotypes in the activation of resolution pathways within human granulosa cells**

Thaise S. Martins^a^, Bruno M. Fonseca^a^, Irene Rebelo^a*^

^a^UCIBIO, REQUIMTE, Department of Biological Sciences, Laboratory of Biochemistry, Faculty of Pharmacy, University of Porto, Porto, Portugal

*Corresponding author: UCIBIO, REQUIMTE, Department of Biological Sciences, Laboratory of Biochemistry, Faculty of Pharmacy, University of Porto. Rua Jorge de Viterbo nº 228, 4050-313, Porto, Portugal. Tel/Fax: +351 220428557; Email: [irebelo@ff.up.pt](mailto:irebelo@ff.up.pt)

**Original blots imagens**

**Figure 4a**

**
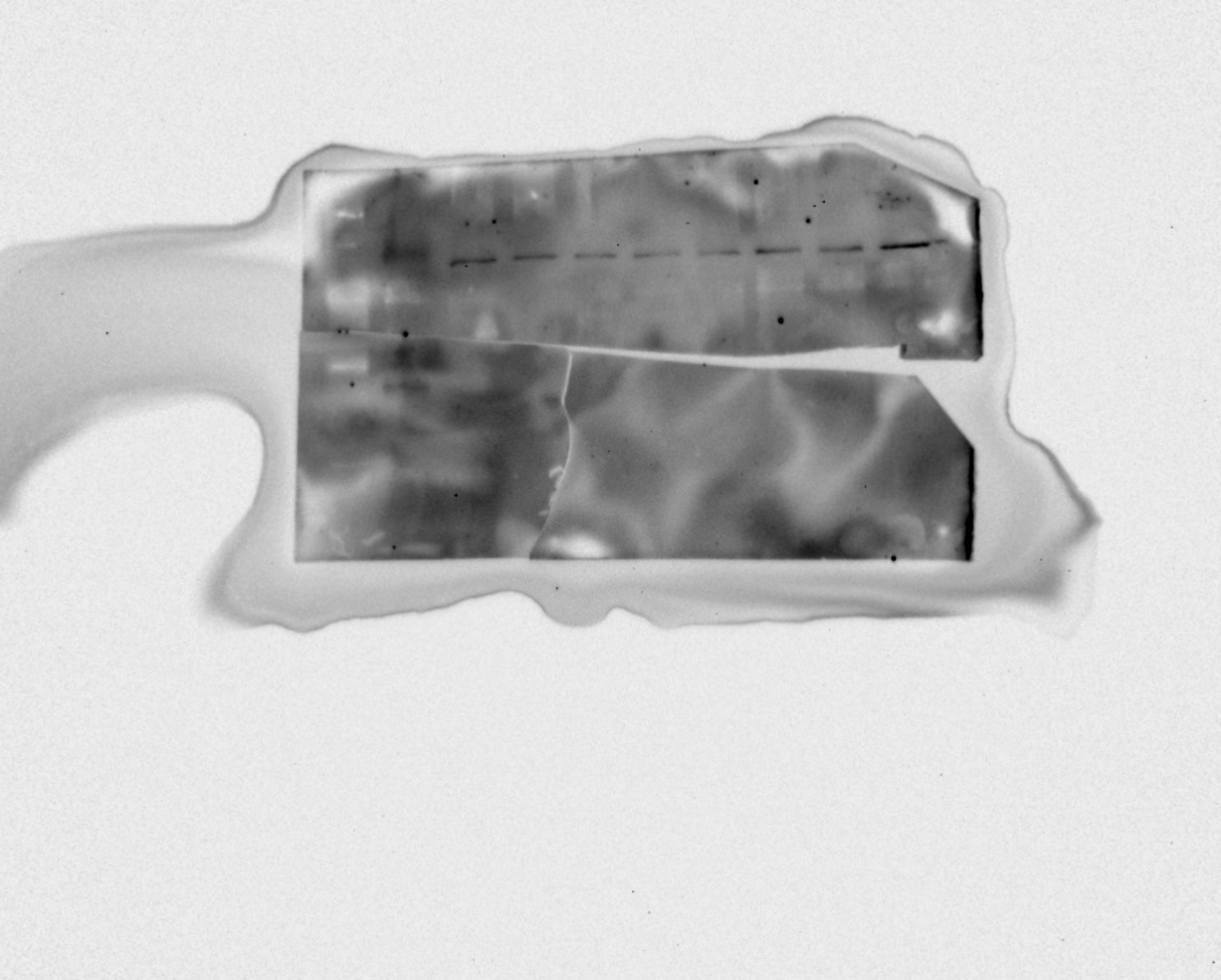
**

COX-2 (75 kDa)

**
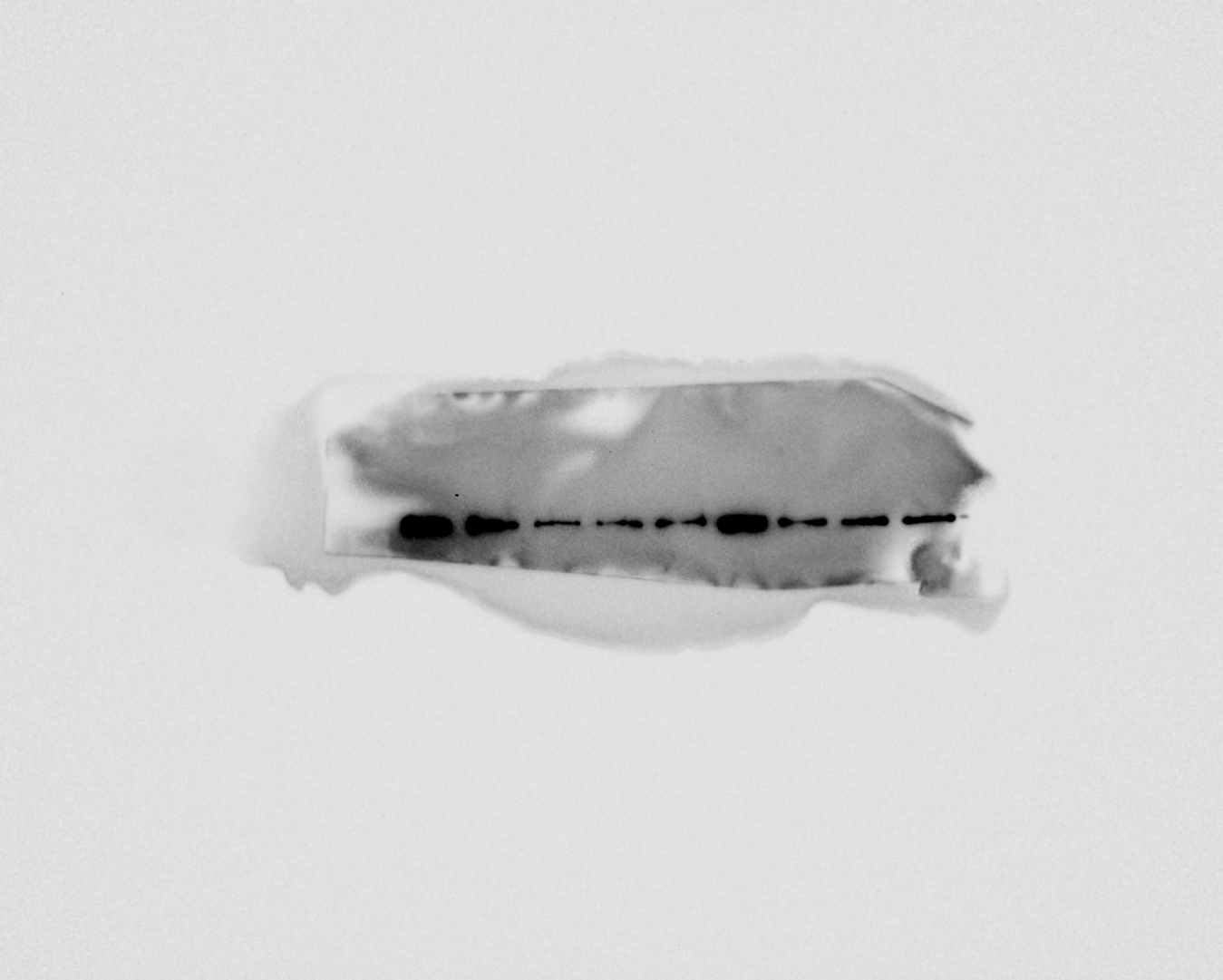
**

β-actin (43 kDa)

**Figure 4b**

**
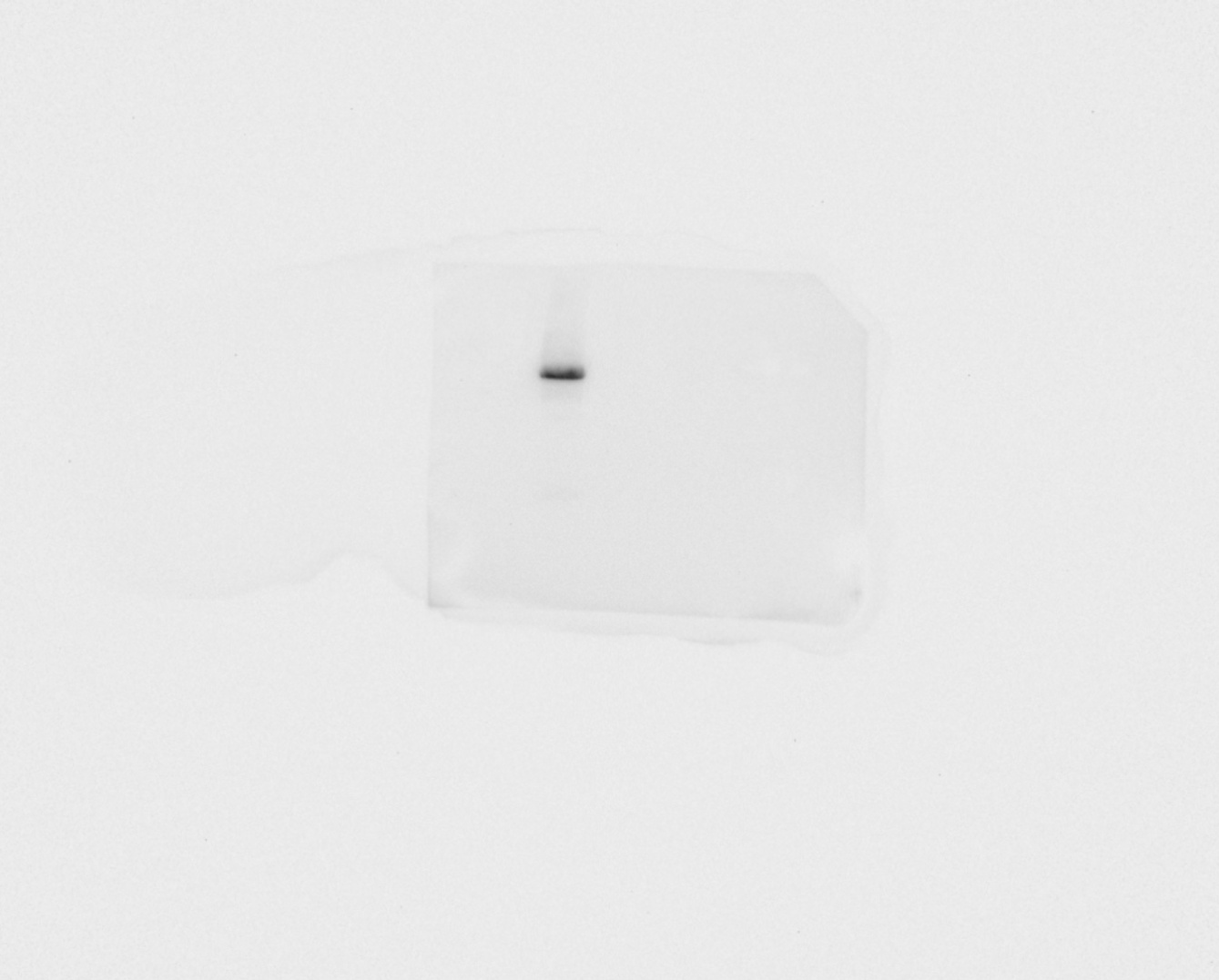
**

5-LOX (70 kDa)

**
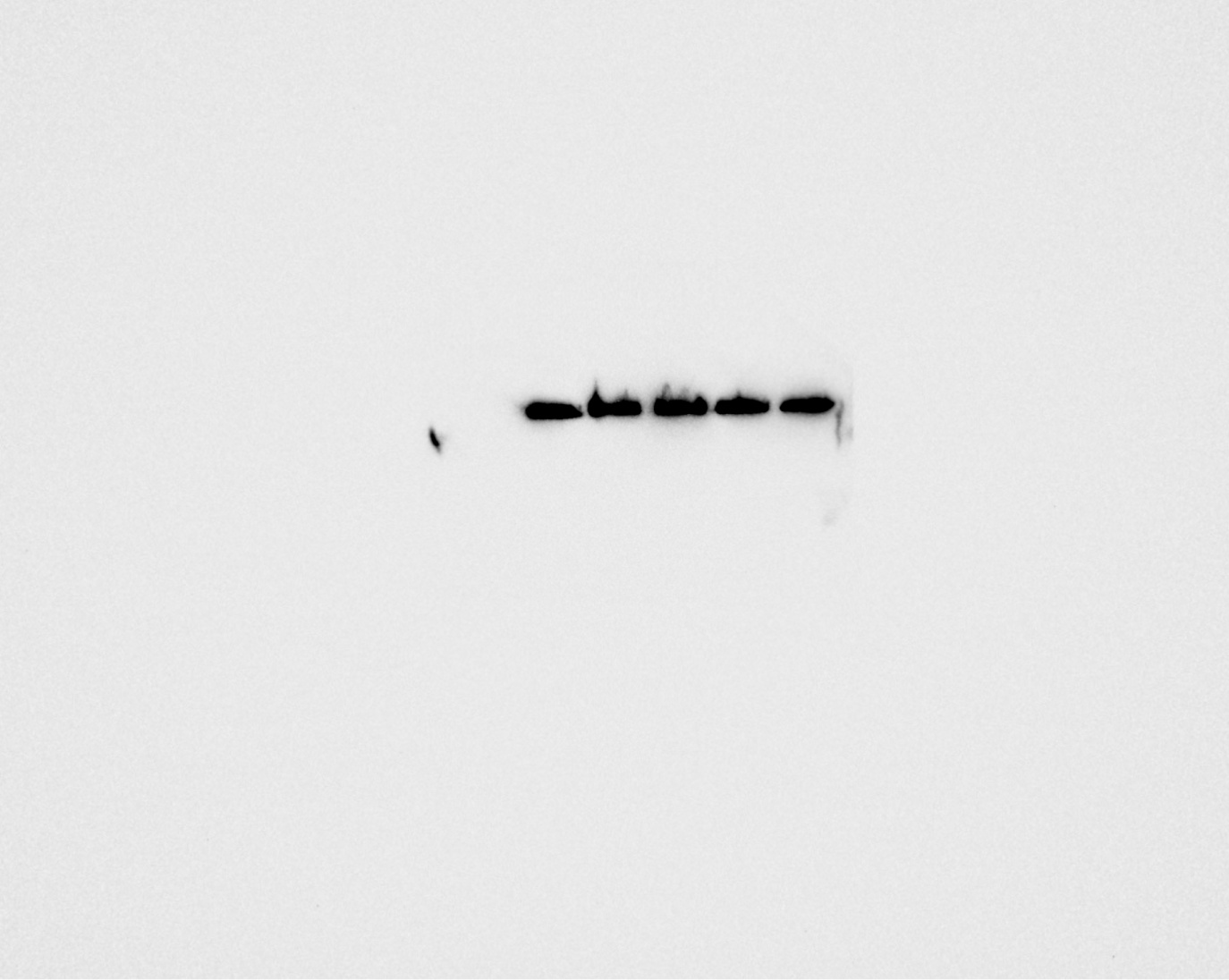
**

β-actin (43 kDa)

**Figure 4c**

**
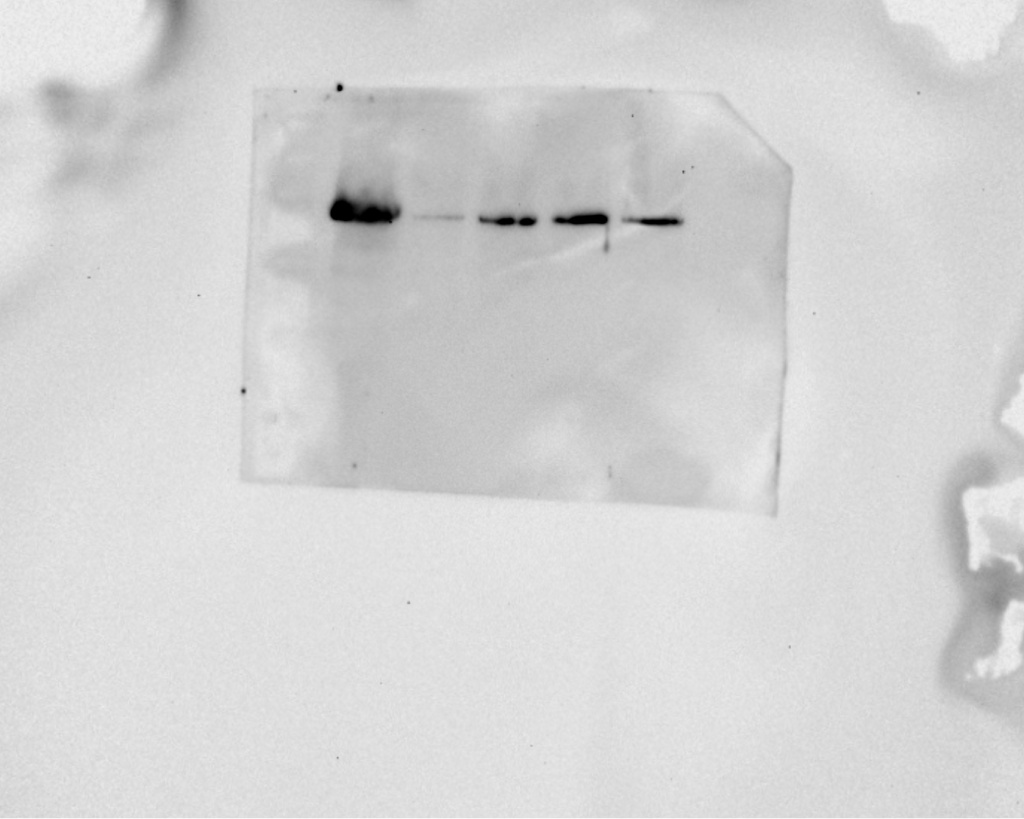
**

12-LOX (70 kDa)

**
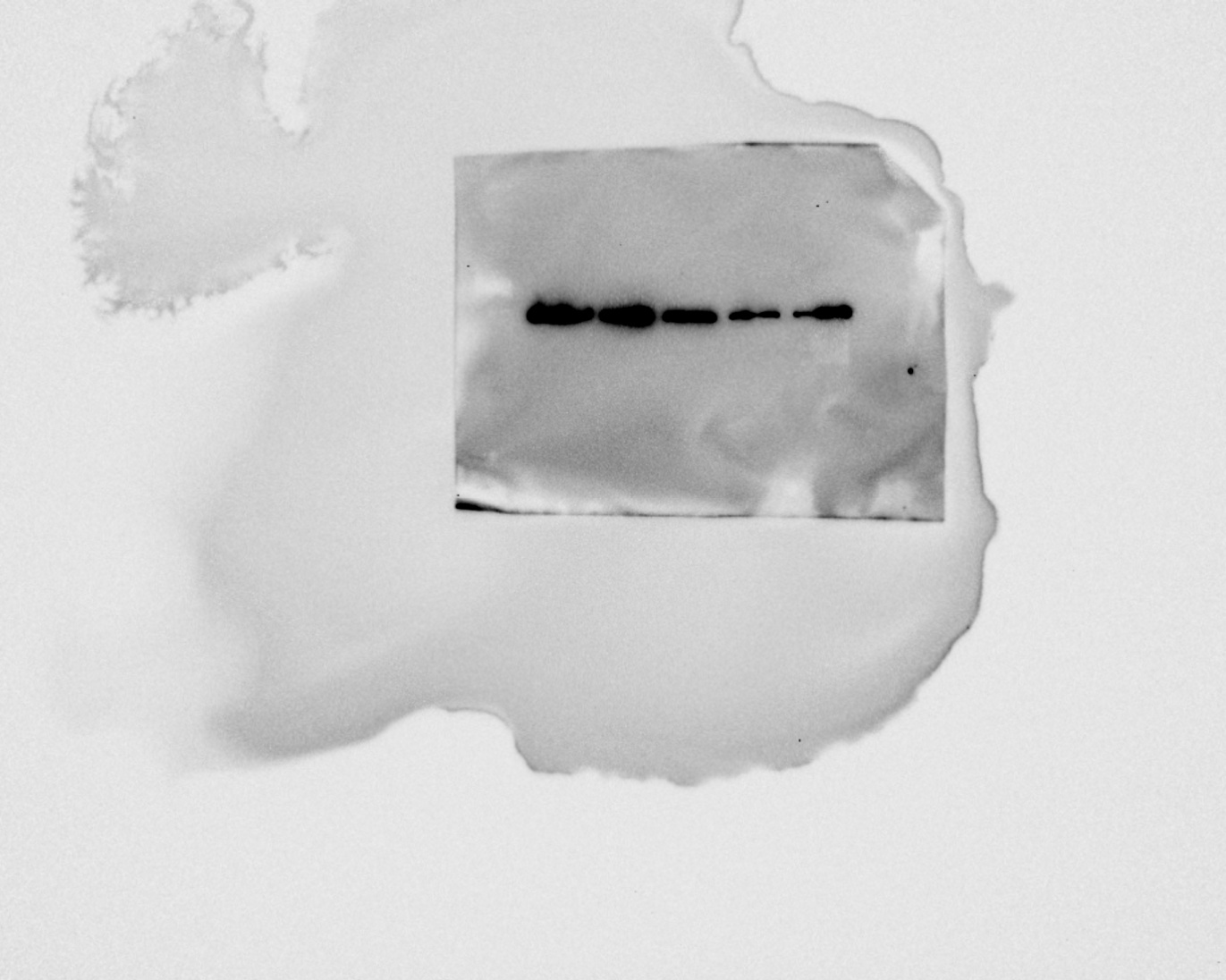
**

β-actin (43 kDa)

**Figure 4d**

**
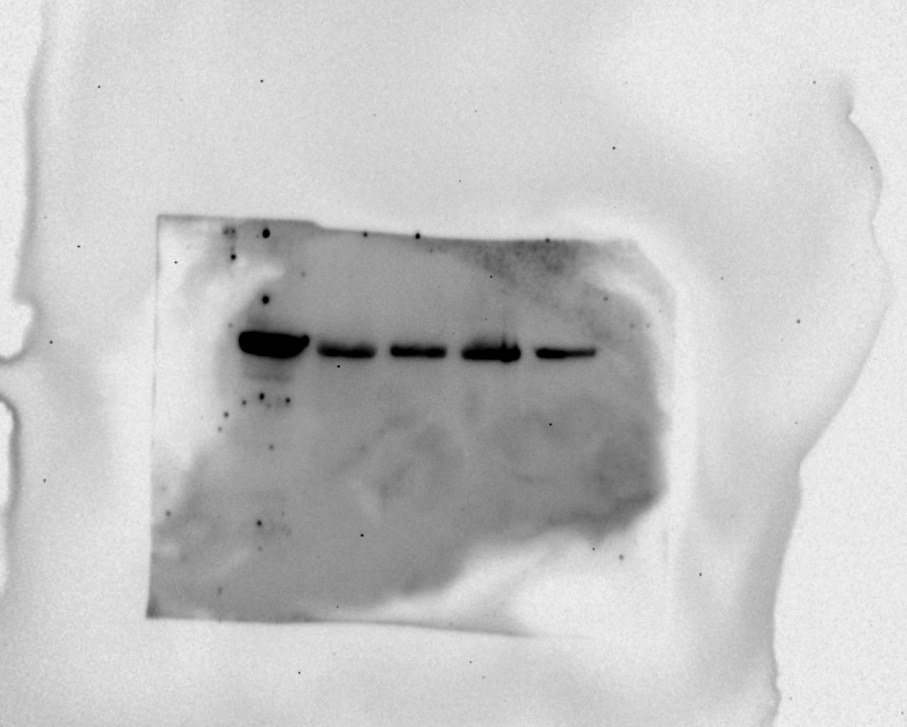
**

15-LOX (75 kDa)

**
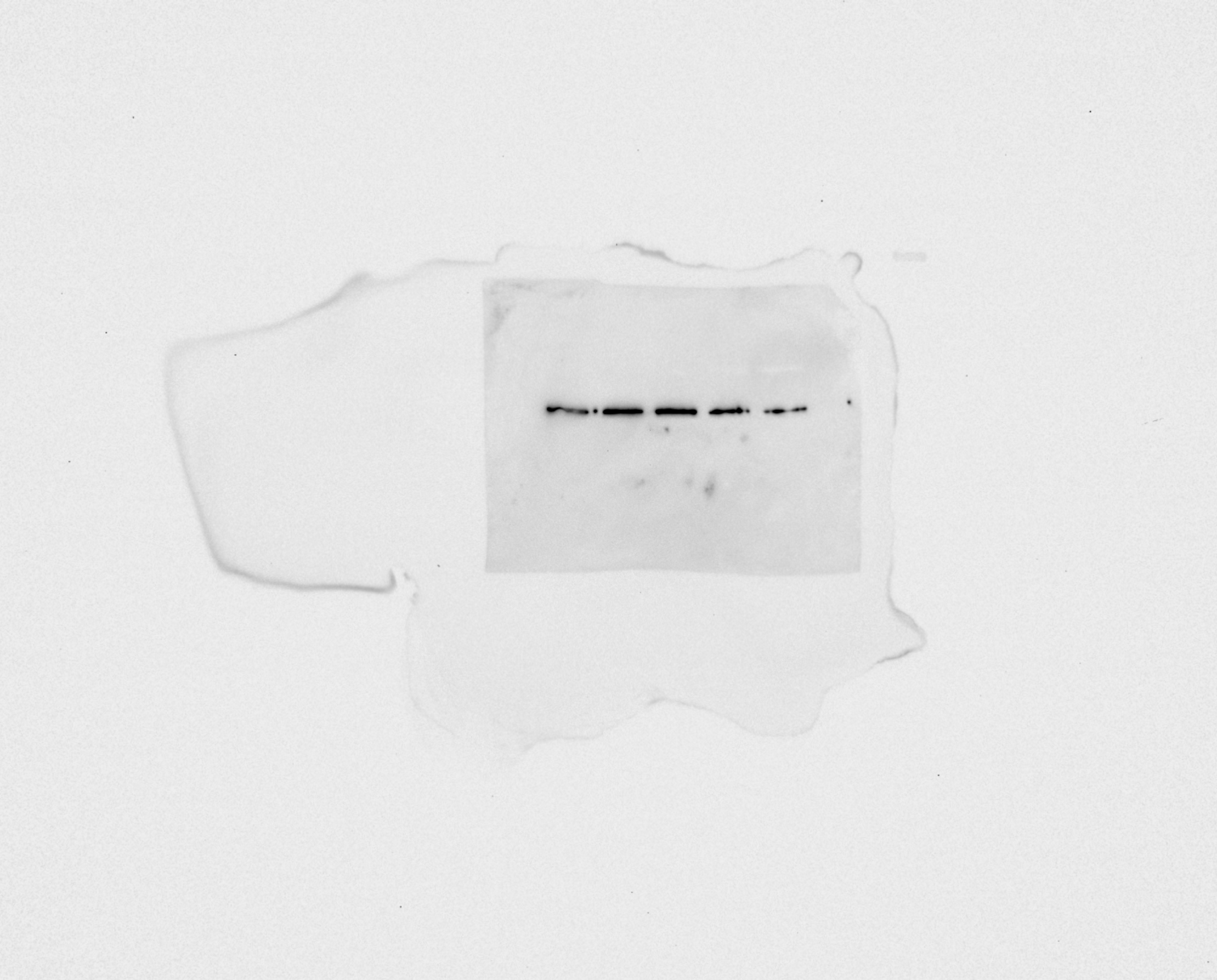
**

β-actin (43 kDa)

**Original blots imagens**

**Figure 5a**

**
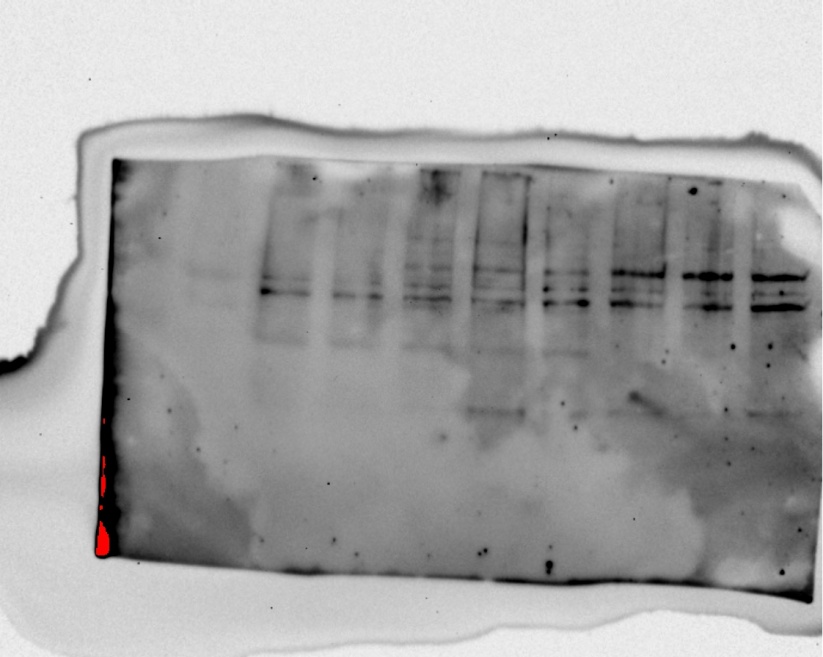
**

COX-2 (75 kDa)

**
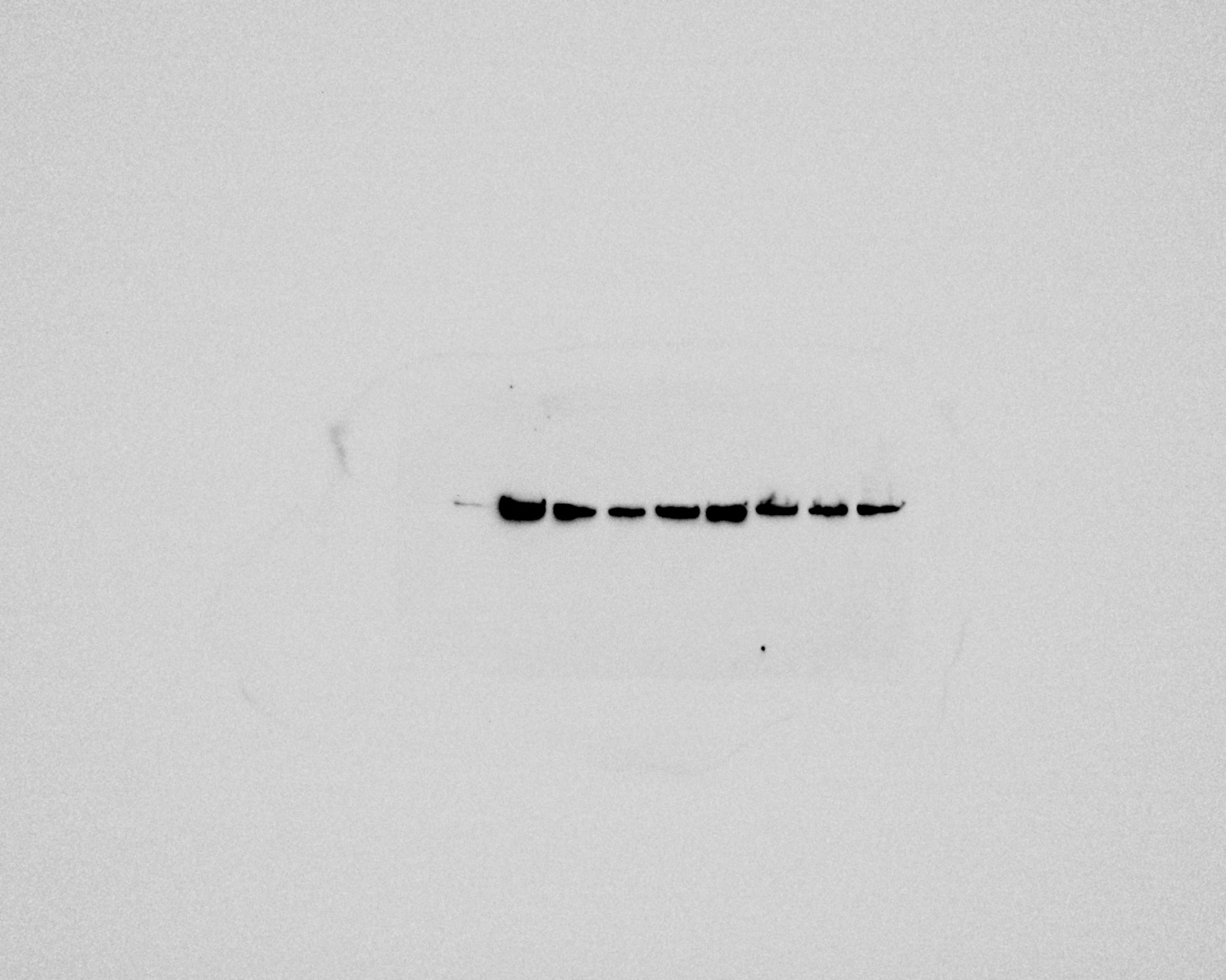
**

β-actin (43 kDa)

**Figure 5b**

**
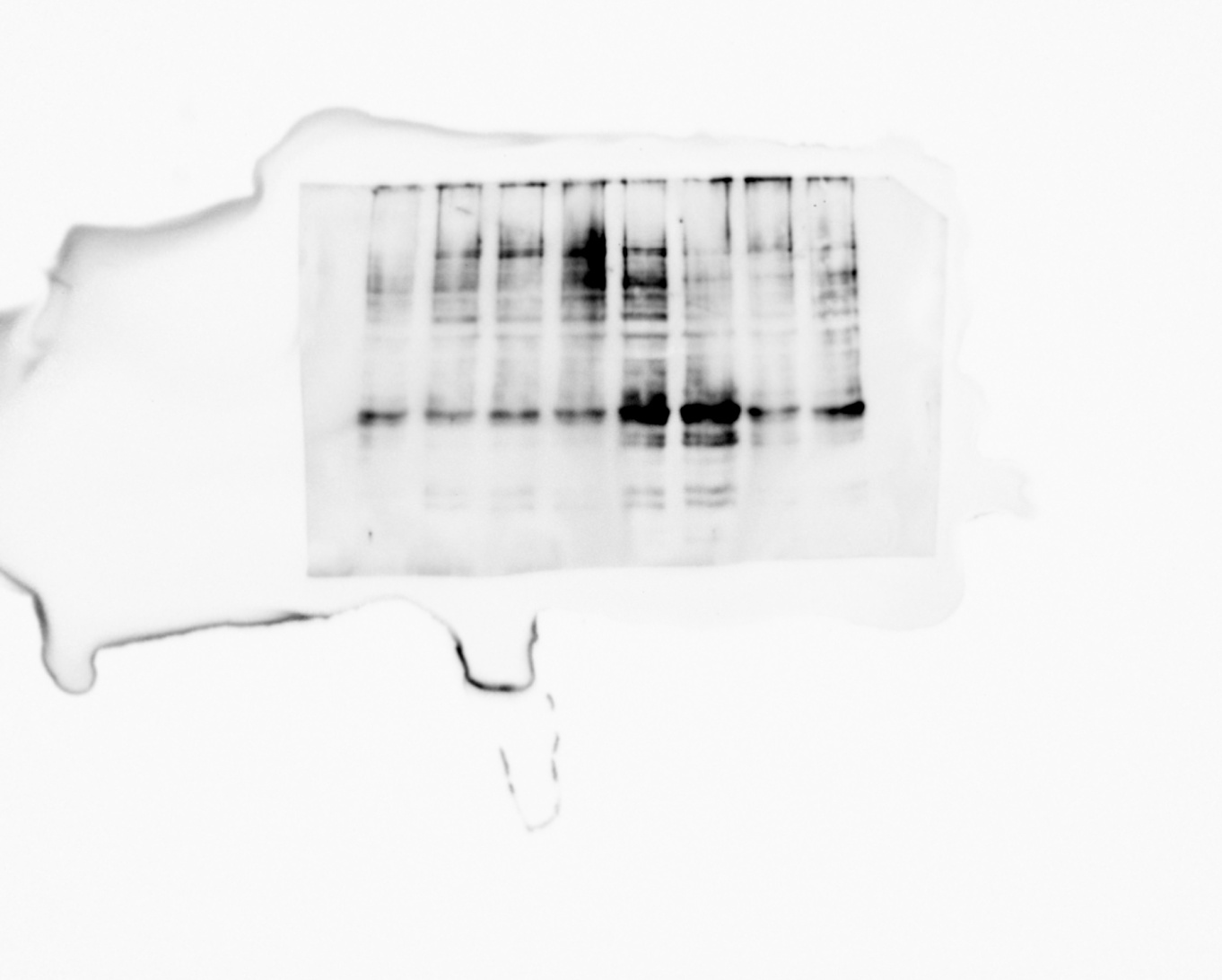
**

5-LOX (70 kDa)

**
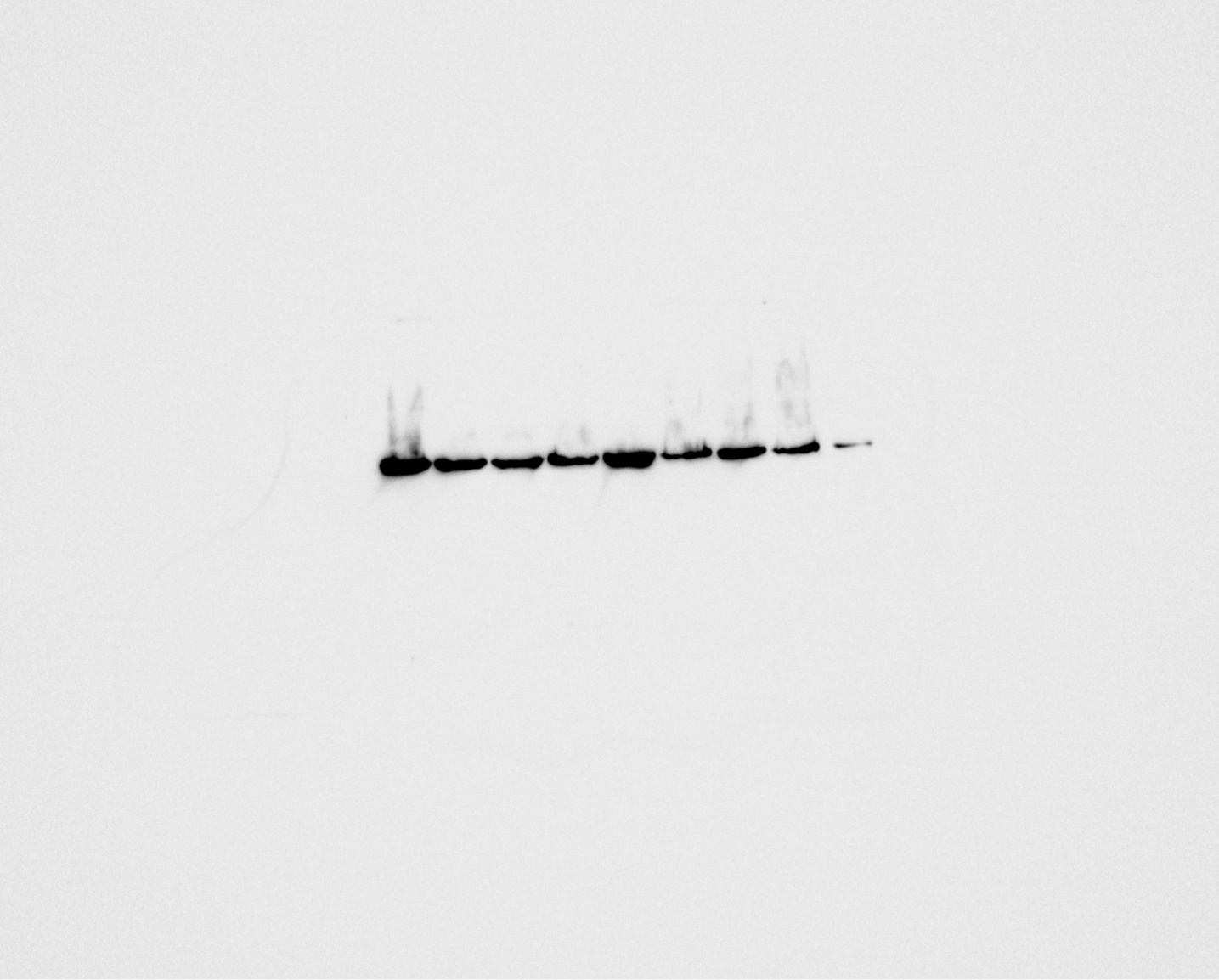
**

β-actin (43 kDa)

**Figure 5c**

**
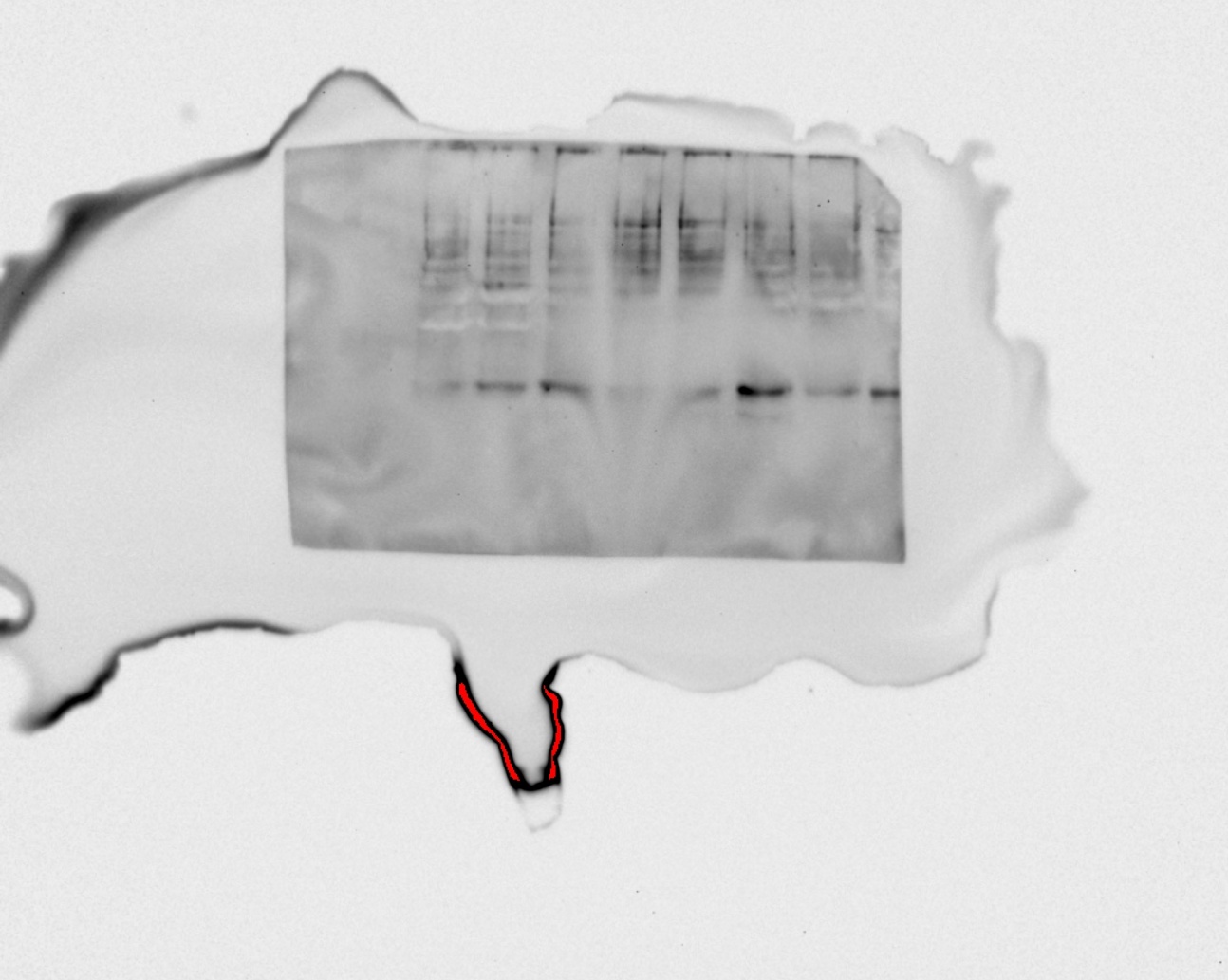
**

12-LOX (70 kDa)

**
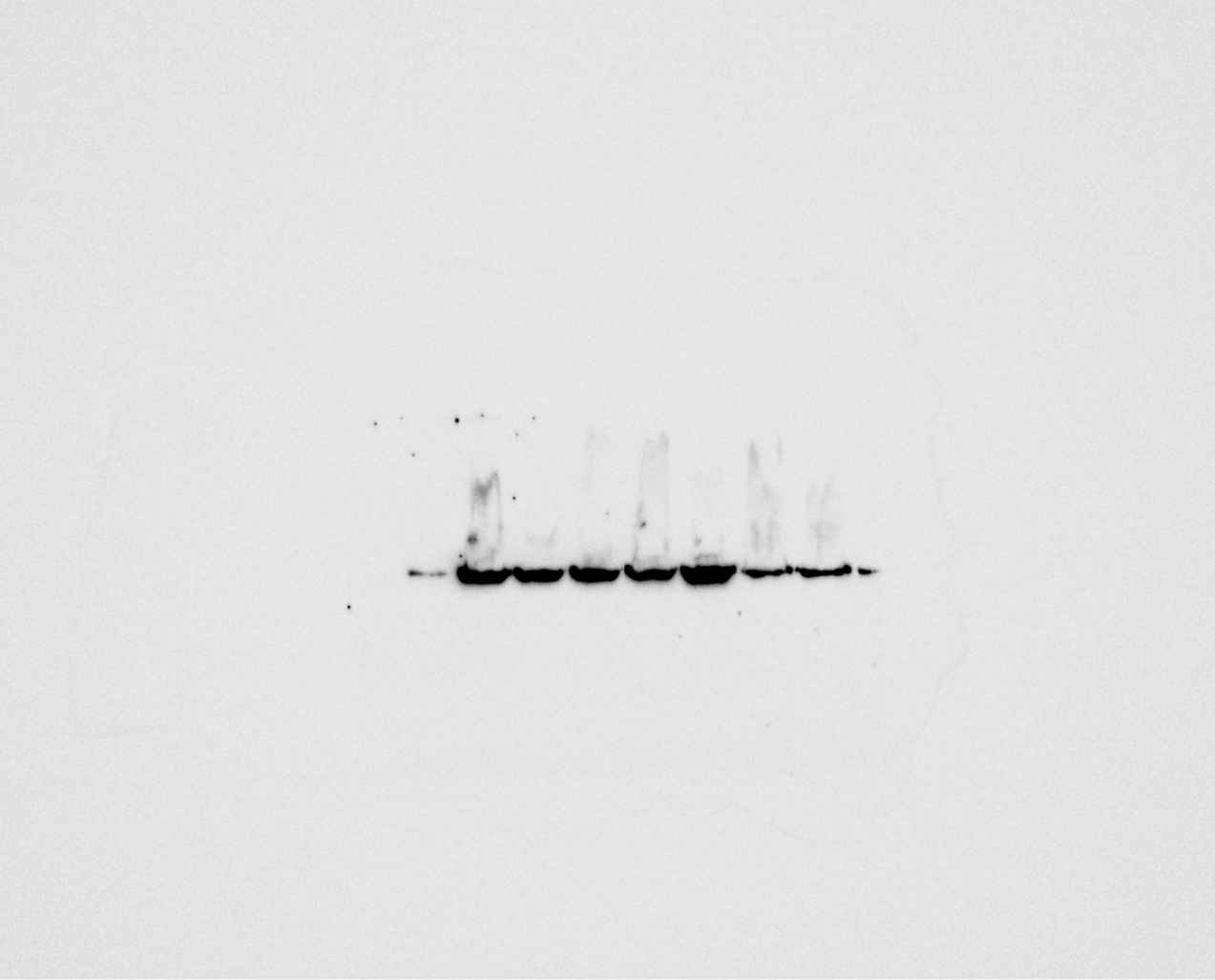
**

β-actin (43 kDa)

**Figure 5d**


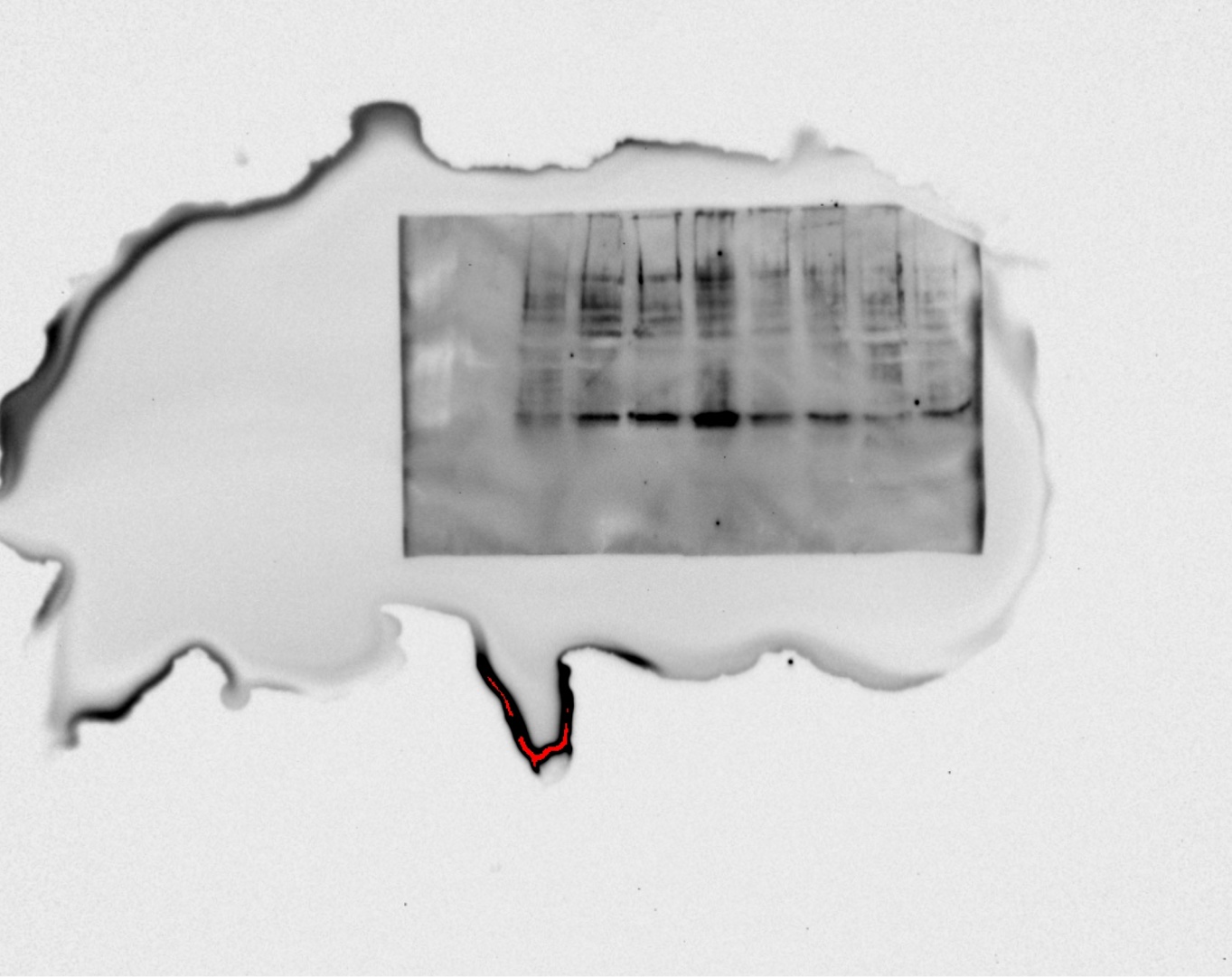


15-LOX (75 kDa)


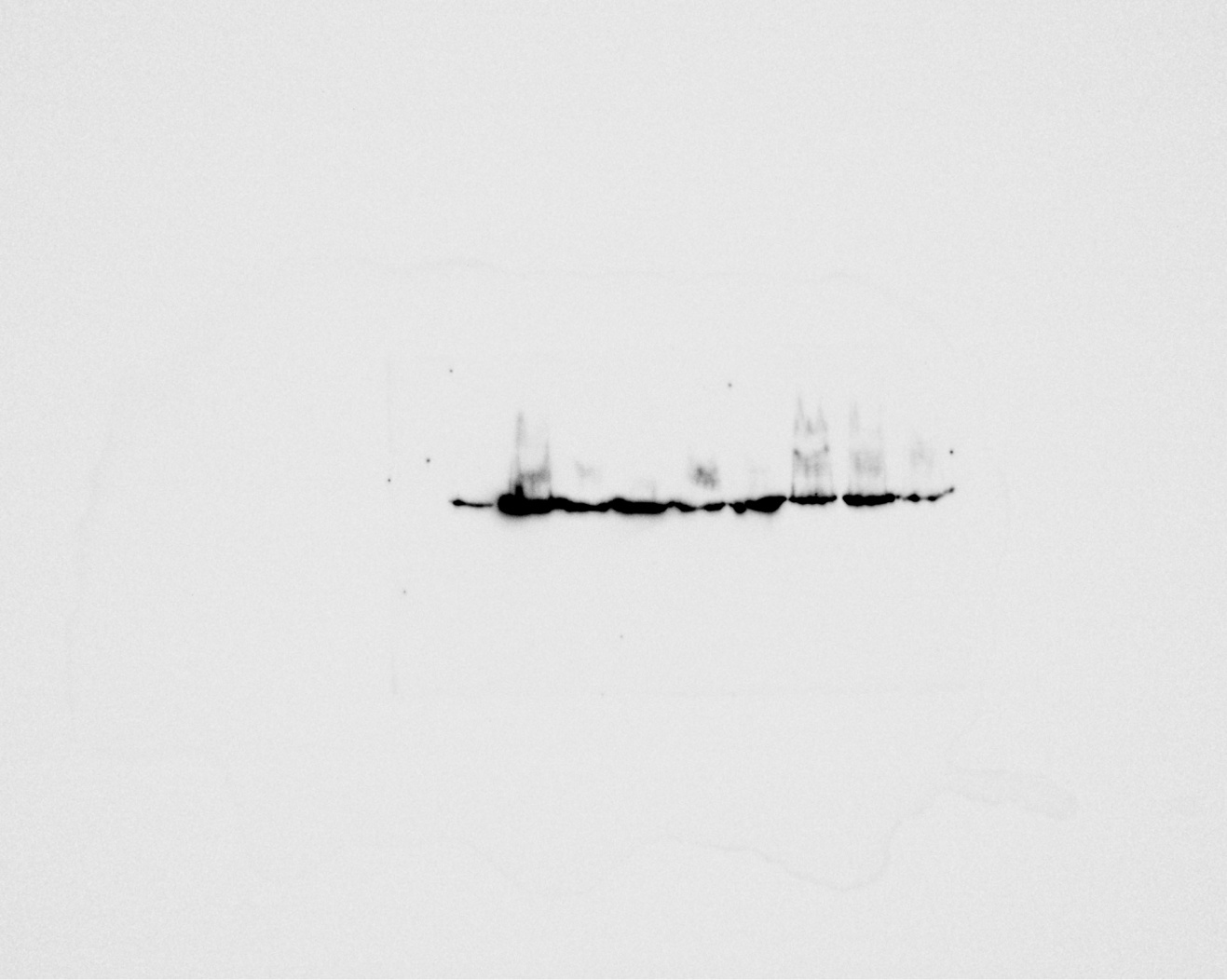


β-actin (43 kDa)

**Supplementary figures 4 and 5:** Uncropped gel from western blots shown in the main figures of the manuscript. Dotted line represents indirect co-culture of conditioned media of M0, M1 and M2 with hGCs (**figure 4**) and COV434 (**figure 5**).
